# Lower Expression of Inducible Nitric Oxide Synthase and Higher Expression of Arginase in Rat Alveolar Macrophages Are Linked to Their Susceptibility to *Toxoplasma gondii* Infection

**DOI:** 10.1371/journal.pone.0063650

**Published:** 2013-05-15

**Authors:** Zhi-Jun Zhao, Jia Zhang, Jun Wei, Zhi Li, Tao Wang, Si-Qi Yi, Ji-Long Shen, Ting-Bao Yang, Geoff Hide, Zhao-Rong Lun

**Affiliations:** 1 State Key Laboratory of Biocontrol, Center for Parasitic Organisms, School of Life Sciences, and Key Laboratory of Tropical Disease and Control (Sun Yat-Sen University), Ministry of Education, Zhongshan School of Medicine, Sun Yat-Sen University, Guangzhou, PR China; 2 General Hospital of Ningxia Medical University, Yinchuan, PR China; 3 Provincial Laboratory of Microbiology and Parasitology and the Key Laboratory of Zoonoses Anhui, Department of Parasitology, Anhui Medical University, Hefei, Anhui, PR China; 4 Ecosystems and Environment Research Centre and Biomedical Research Centre, School of Environment and Life Sciences, University of Salford, Salford, United Kingdom; St. Jude Children’s Research Hospital, United States of America

## Abstract

Rats are naturally resistant to *Toxoplasma gondii* infection, particularly the RH strain, while mice are not. Previous studies have demonstrated that inducible nitric oxide synthase (iNOS) and arginase-1 of rodent peritoneal macrophages are linked to the mechanism of resistance. As an increasing number of studies on human and animal infections are showing that pulmonary toxoplasmosis is one of the most severe clinical signs from *T. gondii* infection, we are interested to know whether *T. gondii* infection in alveolar macrophages of rats is also linked to the levels of iNOS and arginase-1 activity. Our results demonstrate that *T. gondii* could grow and proliferate in rat alveolar macrophages, both *in vitro* and *in vivo*, at levels higher than resistant rat peritoneal macrophages and at comparable levels to sensitive mouse peritoneal macrophages. Lower activity and expression levels of iNOS and higher activity and expression levels of arginase-1 in rat alveolar macrophages were found to be linked to the susceptibility of *T. gondii* infection in these cells. These novel findings could aid a better understanding of the pathogenesis of clinical pulmonary toxoplasmosis in humans and domestic animals.

## Introduction

*Toxoplasma gondii* is an obligate intracellular parasitic protozoan causing toxoplasmosis in infected humans and animals. In most cases, *T. gondii* causes asymptomatic infection in healthy individuals, but severe clinical presentations can be found in congenital toxoplasmosis, ocular toxoplasmosis and in immunocompromised people including AIDS patients [1]. Pulmonary toxoplasmosis has been reported from immunocompromised or immunodeficient patients [2]–[10], pregnant women [11] and immunocompetent individuals [12]–[17]. Additionally, animals with toxoplasmic pneumonia have also been reported in a large number of studies [18]–[21]. However, little attention has been focused on this disease, due to the difficulties of diagnosis, leading to the reporting of relatively few cases [6], [22]. It has been recognized that the lungs are one of the most susceptible organs (following the CNS) to *T. gondii* infection [23] and there are considerable concerns especially when considering lung transplantation [24].

Alveolar macrophages are one of the most important components of the first line of pulmonary defense against inhaled pathogens and other microorganisms [25]–[28]. Yet, controversy still surrounds the characteristics of alveolar macrophages infected with *T. gondii* [5], [29]. Chinchilla and colleagues considered that macrophages are important in the mechanism of resistance to *T. gondii* infection although they did not know what the mechanism was [29]. The mechanism of innate resistance to *Toxoplasma* infection in macrophages was suggested to be a non-oxidative mechanism [30], [31] and was considered to be related to IFN-γ, TNF-α, IL-12, IL-10, TGF-*β* and other cytokines [32]. The function of nitric oxide (NO) and arginine in mouse macrophages against pathogen infection has been well documented [33]–[42]. In fact, it is well known that the L-arginine metabolic pathway plays an important role in host defense and the control of inflammatory reactions [43]–[45]. The relationship between NO and L-arginine is integrally linked. The enzyme which produces NO, inducible nitric oxide synthase (iNOS), utilizes L-arginine as a substrate. However, it also competes with the enzyme, arginase-1, for L-arginine as a substrate. Arginase-1 hydrolyzes L-arginine to L-ornithine and urea. L-ornithine promotes parasite growth as it is a precursor for a variety of polyamines via the ornithine decarboxylase (ODC) pathway [46]–[48]. Thus the balance between iNOS expression (pathogen destruction) and arginase-1 expression (pathogen promotion) is postulated to be linked to susceptibility or resistance to *Toxoplasma* infection.

In our previous work, we demonstrated that the differences in expression levels and activity of iNOS and arginase-1 between the rat peritoneal macrophages (RPMs) and mouse peritoneal macrophages (MPMs) are strongly linked to the resistance and host specificity for *Toxoplasma* infection in these cells and hosts [49]. As severe clinical signs are commonly observed in the lungs of the host infected with *T. gondii*, we were interested to know if the infectivity of the rat alveolar macrophages (RAMs) for *T. gondii* is similar to those from the peritoneal cavity. The answer to this question could significantly benefit our understanding of the mechanism of pulmonary toxoplasmosis in humans and in domestic animals.

## Results

### Proliferation of *T. gondii* in Rat Alveolar Macrophages

**Figure 1:**
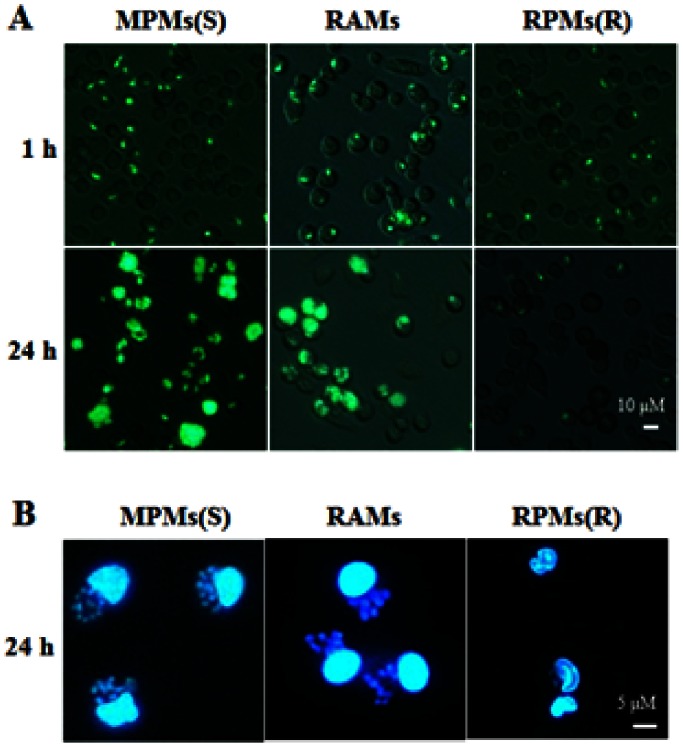
Analysis of *T. gondii* proliferation in rat alveolar macrophages in vitro. Rat alveolar macrophages (RAMs) were incubated with *T. gondii* at the ratios of 1∶1 (parasites/macrophages = 1∶1), then the extracellular *T. gondii* (the non-invaded individuals) were washed from the medium and the time was defined as 1 hr. After 24 hrs, cells from the same cultures were compared. Mouse peritoneal macrophages (MPMs) (susceptible) and rat peritoneal macrophages (RPMs) (resistant) were designated as controls in the infection of *T. gondii*. (A and B) Different methods of Fluorescence Microscopy or DAPI staining were used in these experiments to observe the infection of *T. gondii* in macrophages. All the results were observed by fluorescence microscopy using a 350 nm exciting wavelength and 495 nm emitting wavelength. The results are representative of three similar experiments.

**Figure 2:**
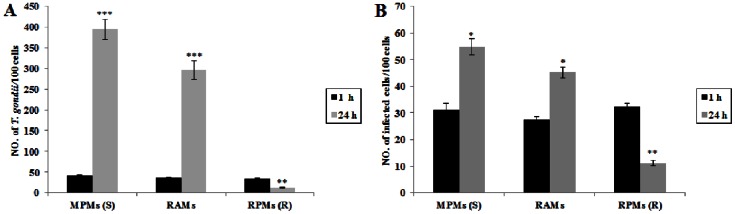
Proliferation of *T. gondii* in RAMs. (A) Number of *T. gondii*/100 cells. (B) Number of infected cells/100 cells. Data in A and B represent a mean ± SEM calculated from 3 independent experiments performed in triplicate. Probabilities *p<0.05, **p<0.01, and ***p<0.001, (ANOVA), indicate a significant difference from infection at 1 hr in MPMs (S), RAMs and RPMs (R) respectively (S: susceptible; R: resistant).

Using infection studies on peritoneal and alveolar macrophages, *T. gondii* was found to be able to multiply in MPMs (sensitive cells) but this proliferation was not seen in RPMs (resistant cells) (Figure 1) as has been reported previously in other studies [28], [49], [50]. However, in rat alveolar macrophages from the same inbred line, a significant proliferation of *T. gondii* was observed and this was similar to the situation seen in the sensitive MPMs (Figure 1). Following infection by *T. gondii*, a significant increase of *T. gondii* in RAMs was found from 1 hr (36.07±1.09 per 100 cells) to 24 hrs after infection (296.44±22.00 per 100 cells) (Figure 2A) when the same population of cells were infected. However, a highly significant decrease in the number of *T. gondii* in RPMs was observed from 1 hr (33.91±1.89 per 100 cells) to 24 hrs (12.79±1.26 per 100 cells) after infection using the same experimental approach. Although the number of *T. gondii* in RAMs was less than that in MPMs at 24 hrs after infection, the massive proliferation of this parasite in RAMs indicated that this cell type is highly susceptible to *T. gondii* infection. Moreover, a significant increase in the ratio of infected to non-infected cells in the RAM samples was also found between 1 hr (27.49±1.27 per 100 cells) and 24 hrs (45.22±1.99 per 100 cells) post infection (Figure 2B). This was similar to the situation observed in MPMs at 1 hr (31.24±2.39 per 100 cells) and 24 hrs (54.92±2.96 per 100 cells) after infection. In addition to reporting the high degree of sensitivity of RAMs, these results also confirmed the results reported in our previous publication that the proportion of infected cells was highly significantly decreased in RPMs from 1 hr (32.28±1.44 per 100 cells) to 24 hrs (11.18±1.15 per 100 cells) after infection [49].

### iNOS/arginase Expression and Activity in Rat Alveolar Macrophages

**Figure 3:**
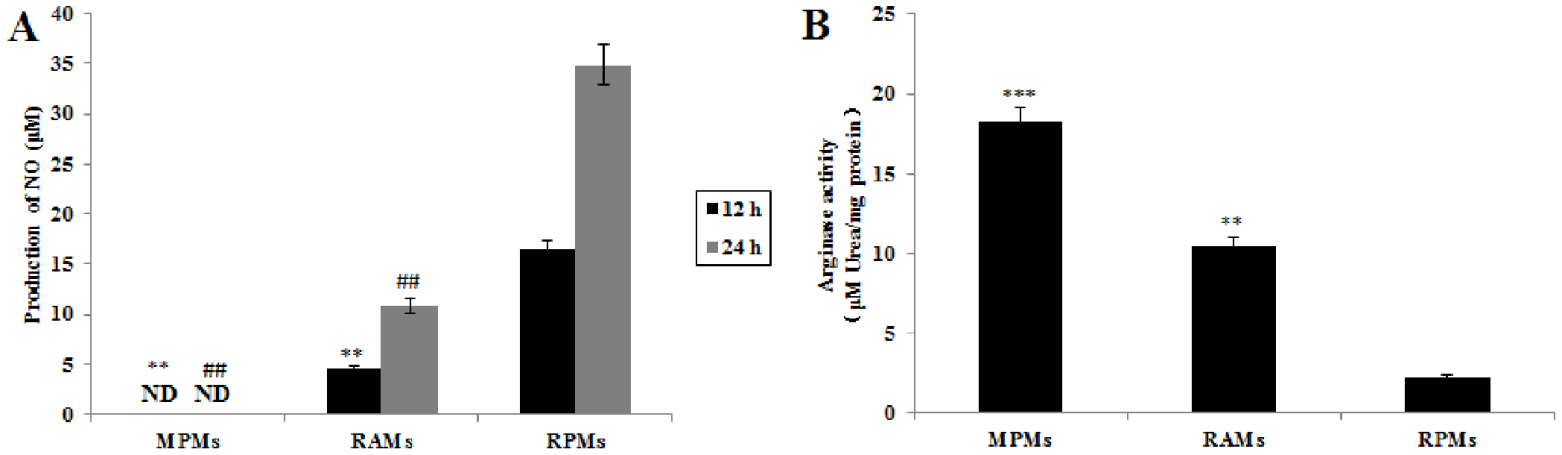
Comparison of nitric oxide production and arginase activity among RAMs, MPMs and RPMs. (A) A comparison of NO production between different macrophage types as measured by the Griess reaction, relative to RPMs. Data represent mean ± SEM calculated from 3 independent experiments performed in triplicate. Probabilities **p<0.01, (ANOVA), show significant differences from NO production of RPMs cultured for 12 hrs; probabilities ^##^ *p*<0.01, (ANOVA), show significant differences from NO production of RPMs cultured for 24 hrs. (B) A comparison of arginase activity, as measured by a colorimetric assay (enzyme activity is the output of urea secreted from lysed macrophages), between different macrophage types relative to RPMs. Data represent a mean ± SEM calculated from 3 independent experiments performed in triplicate. Probabilities **p<0.01 and ***p<0.001, (ANOVA), show significant differences from arginase activity of freshly purified RPMs.

**Figure 4:**
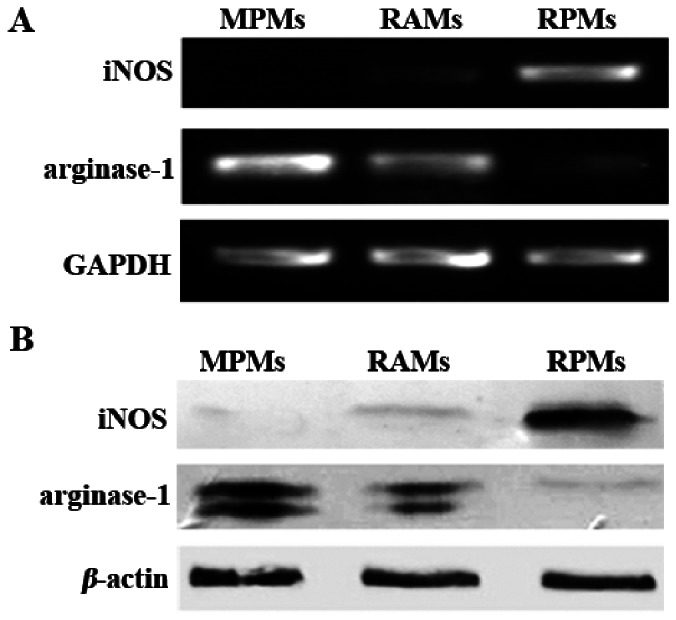
iNOS/arginase expression in MPMs, RAMs and RPMs. (A) RT-PCR analysis for the expression of iNOS and arginase-1 mRNA in MPMs, RAMs, and RPMs, using a housekeeping gene, GAPDH, as a control. (B) Western-blotting analysis of the expression of iNOS and arginase-1 protein in MPMs, RAMs, and RPMs, using a housekeeping protein, *β*-actin, as a control. The results are representative of three similar experiments.

Since iNOS and arginase are competitors for the same substrate (arginine) in the L-arginine metabolic pathway, we analyzed the level of iNOS/arginase activity and gene expression in non-activated alveolar macrophages freshly isolated from rats. High iNOS/low arginase activity and expression were found in RPMs, while lower iNOS/higher arginase activity and expression were found in RAMs. As a matter of fact, these levels were similar to those found in the sensitive MPMs. Although RAMs could secrete a small amount of NO (in comparison with the non-detectable NO production in MPMs), the concentration of NO production (10.89±0.73 µM) was significantly lower than RPMs (34.86±1.96 µM) especially at 24 hrs after cultivation *in vitro* (Figure 3A). When comparing the arginase activity between rat peritoneal and alveolar macrophages, our results showed that arginase activity of RAMs (10.47±1.72 µM urea/mg protein) was much higher than that found in RPMs (2.26±0.25 µM urea/mg protein), but lower than that found in MPMs (18.21±1.87 µM urea/mg protein) as the sensitive control (Figure 3B). To investigate whether these differences in activity levels are due to differences in expression of the iNOS and arginase-1 genes, mRNA and protein levels were measured. Our results showed that the level of iNOS mRNA expression in RAMs was much lower than that in RPMs. In contrast, the mRNA expression of arginase-1 was highly expressed in RAMs (Figure 4A). Results from Western-blot analysis also indicated that iNOS protein expression in RAMs was much less than that found in RPMs, while higher expression of arginase-1 protein expression was found in RAMs (Figure 4B).

### Changes in NO Production in RAMs and Response to Infection with *T. gondii*

**Figure 5:**
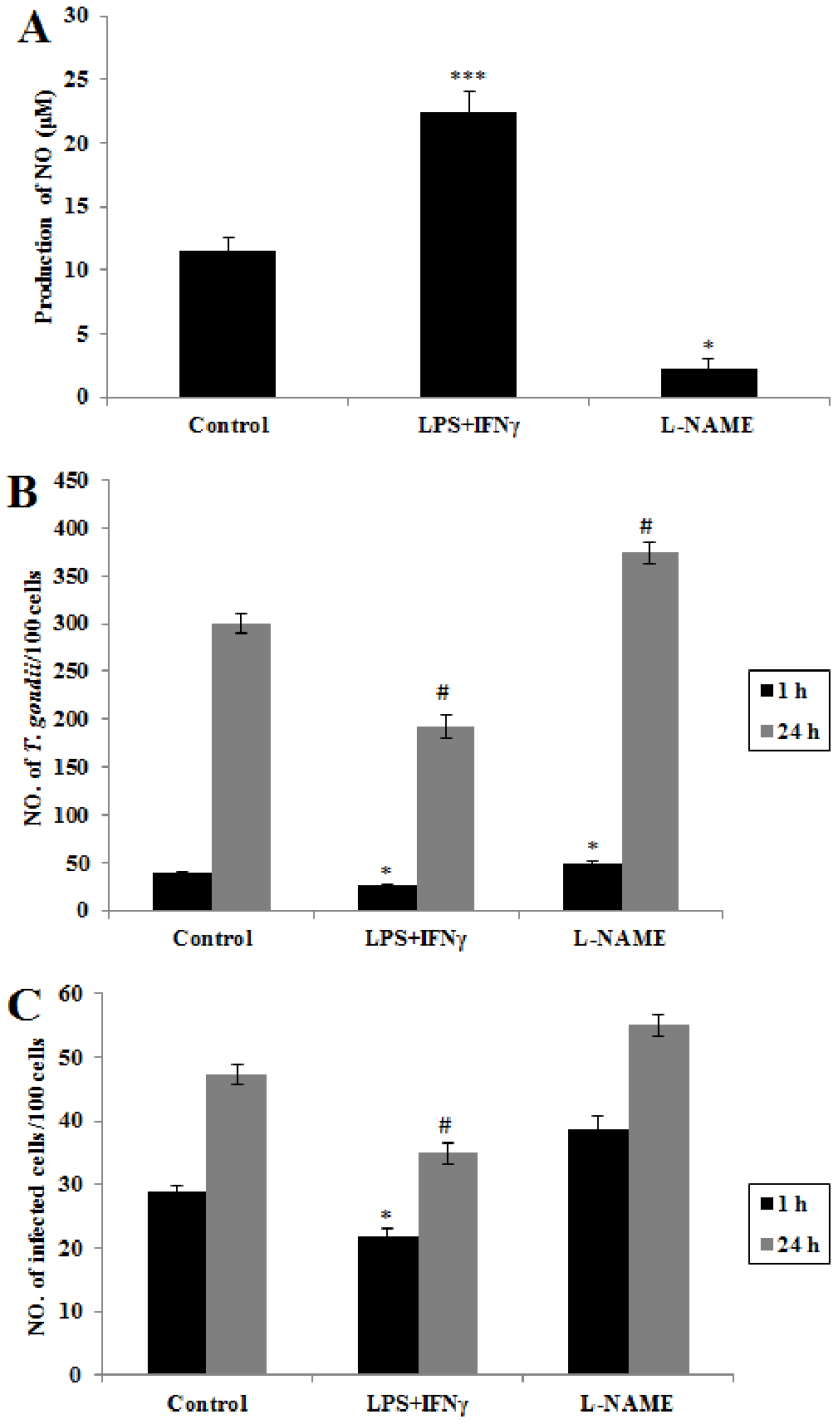
Effect of NO regulation on the growth of *T. gondii* in rat alveolar macrophages. (A) RAMs were treated with LPS+IFN-γ or L-NAME for 12 hrs. NO production of each group was measured by the Griess reaction, using normal cultured RAMs as control. Data represent a mean ± SEM calculated from 3 independent experiments performed in triplicate. Probabilities *p<0.05 and ***p<0.01, (ANOVA), show significant differences from controls. (B) Comparison of number of *T. gondii* per 100 cells. (C) Comparison of number of infected cells per 100 cells. Data in B and C represent a mean ± SEM calculated from 3 independent experiments performed in triplicate. Probabilities *p<0.05 and ^#^ *p*<0.05, (ANOVA), show significant differences from controls of infection at 1 hr and 24 hrs respectively. ND: not detectable.

Since MPMs were susceptible to *T. gondii* RH strain infection and the major reason was due to the lack of NO production [49], we wanted to investigate the growth of *T. gondii* in RAMs stimulated by LPS+IFN-γ (stimulators of NO production) and inhibited by L-NAME. Figure 5A shows that NO production was significantly increased in the cells treated with LPS+IFN-γ but decreased in the macrophages treated with L-NAME. We further demonstrate that the number of *T. gondii*/100 cells was significantly decreased in the macrophages treated with LPS+IFN-γ but significantly increased in those treated with L-NAME (*p*<0.05) at 24 hrs after infection (Figure 5B). The profile of the ratio of infected cells to uninfected cells showed a similar significant pattern (Figure 5C).

### The Growth of *T. gondii* in Alveolar Macrophages from Different Strains of Rats *in vitro*

**Figure 6:**
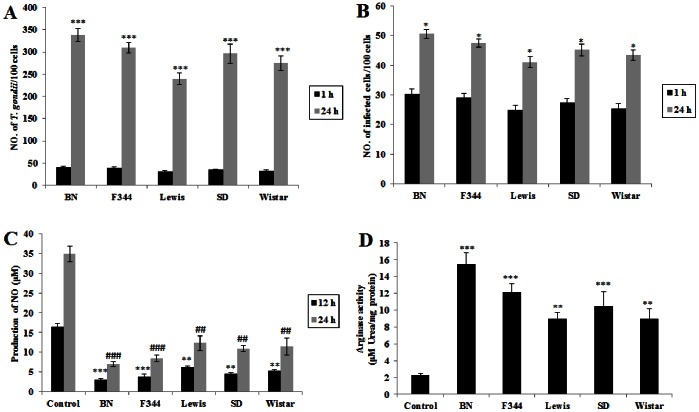
Growth status, production of NO and arginase activity of *T. gondii* in alveolar macrophages isolated from different strains of rats (BN, F344, Lewis, SD and Wistar). Rat alveolar macrophages (RAMs) were incubated with *T. gondii* at the ratios of 1∶1 (parasite/macrophages = 1∶1), then the extracellular *T. gondii* (the non-invaded individuals) were washed from the medium and the time was defined as 1 hr. The infection results were observed by fluorescence microscopy. (A) Comparison of number of *T. gondii* per 100 cells. (B) Comparison of number of infected cells per 100 cells. Data in A and B represents a mean ± SEM calculated from 3 independent experiments performed in triplicate. Probabilities *p<0.05 and ***p<0.001, (ANOVA), show significant differences from the infection at 1 hr of each type of macrophage respectively. (C) NO production of different rat alveolar macrophages was measured by the Griess reaction. Data represent a mean ± SEM calculated from 3 independent experiments performed in triplicate. Probabilities **p<0.01 and ***p<0.001, (ANOVA), show significant differences from NO production of control (RPMs cultured for 12 h); probabilities ^##^ *p*<0.01 and ^###^ *p*<0.001, (ANOVA), show significant differences from NO production of control (RPMs cultured for 24 h). Control: SD rat peritoneral macrophage. (D) Arginase activity of different rat alveolar macrophages were measured by a colorimetric assay (enzyme activity is the output of urea secreted from lysed macrophages). Data represent mean ± SEM calculated from 3 independent experiments performed in triplicate. Probabilities **p<0.01 and ***p<0.001, (ANOVA), show significant difference from control (SD rat peritoneal macrophages).

Previous studies [49] showed that there was variation in iNOS/arginase-1 activity in different inbred lines of rats suggesting genetic control of host enzyme activity and therefore degree of susceptibility to *T. gondii* infection. Figure 6A and 6B show that the levels of *T. gondii* infection in alveolar macrophages isolated from different inbred lines of rats (BN, F344, Lewis, SD and Wistar) differ between strains. These results also demonstrate that sensitivity to *T. gondii* infection by rat alveolar macrophages is not confined to individual inbred lines and therefore suggests a common response in rats. Figure 6C shows that the NO production in non-activated alveolar macrophages from different strains of rats was significantly lower than the controls (RPMs). However, the arginase activity of alveolar macrophages from different strains of rats was significantly higher than the controls (RPMs) (Figure 6D). The susceptibility of these populations of RAMs from different inbred lines of rats, as measured by infection levels, was completely correlated with arginase-1 activity and inversely correlated with NO production.

### Infection of Rat Alveolar Macrophages *in vivo* by *T. gondii*

**Figure 7:**
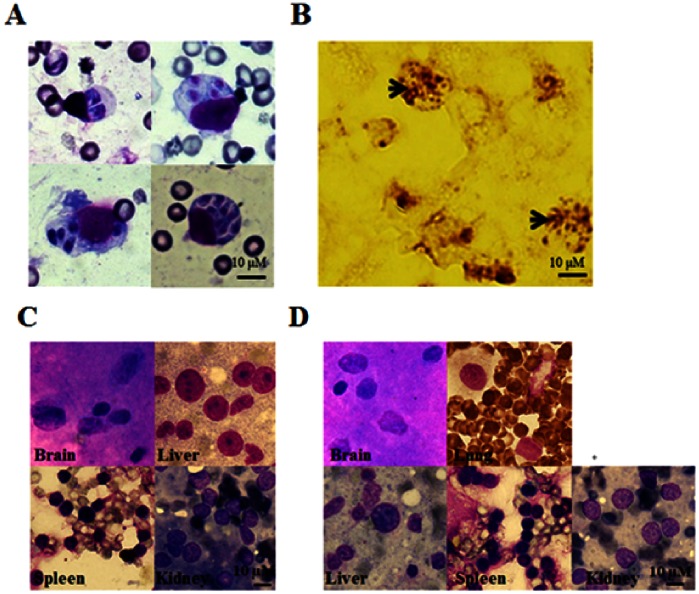
Analysis of *T. gondii* infection in rat alveolar macrophages in vivo. Rats were infected by pulmonary infection (n = 6); peritoneal infection (n = 6) and uninfected controls (n = 6; injected with PBS) to determine whether *T. gondii* could be taken up in vivo. (A) At 24 hrs after the time of infection, the lungs of infected rats were sectioned, directly smeared and then stained with Diff. The results are representative of the replicated experiments. (B) Similarly, the lungs of these infected rats were steeped in 10% polyphosphate formalin for 24–48 hrs, then treated by immunohistochemistry with an specific anti-toxoplasma antibody. The arrows indicate the proliferation of *T. gondii* in RAMs *in vivo*. The results were observed by microscopy (100×). The results are representative of the replicated experiments. (C) Diff stained section of brain, liver, spleen and kidney tissue following pulmonary infection showing that parasites could only be found in alveolar macrophages and not in other tissues. The results are representative of the replicated experiments (6 rats per experiment). (D) Diff stained section of brain, lung, liver, spleen and kidney tissue following peritoneal infection showing that parasites could not be found in any tissues. The results are representative of the replicated experiments (n = 6).

Although we have demonstrated that RAMs are susceptible to *T. gondii* infection *in vitro*, we did not know if similar results could be confirmed *in vivo.* Groups of 6 rats were infected by either the pulmonary route, peritoneal route or were not infected (injected with PBS). Figure 7A shows that we can detect the infection of *T. gondii* in alveolar macrophages of SD and Wistar rats infected directly from the pulmonary cavity. Different numbers of tachyzoites were observed within cells which could have been generated either by differential infection with *T. gondii* cells or by post-infection intracellular proliferation. Immunohistochemical tests also confirmed the presence of parasites in the alveolar macrophages (Figure 7B). Tachyzoites were not detected in other organs of these rats which had been infected directly via the pulmonary cavity (Figure 7C) demonstrating that the rat lungs were more susceptible to the parasites. To clarify this, parasites were infected peritoneally, using both SD and Wistar rats, but no parasites were found in any organs (brain, lung, liver, spleen and kidney) in these infected rats (Figure 7D). This confirms that infection *in vivo* is particularly occurring in the RAMs and not, as has been previously observed, in RPMs. These results show that the *in vitro* results, on susceptibility of RAMs and resistance of RPMs, are also observed *in vivo*. These data also show that intraperitoneal infection in rats does not appear to spread, even to lung tissue at least in the case of the *T. gondii* RH strain. While pulmonary injection results in infection of RAMs but does not appear to, under the conditions used here, spread systemically. Thus, different outcomes of infection can be observed when using the two routes of inoculation. It would be an interesting question to investigate the mechanisms of cell/macrophage invasion during the natural (fecal-oral) infection route. However, it is not suitable for this study because of the complicated life cycle of *T. gondii* and variability in the efficiency of infection of the host when infected in this way. In addition, technical difficulties would need to be overcome to achieve reproducibility in the rat due to resistance to *T. gondii* and difficulties in ensuring accurate and comparative delivery doses of oocysts or cysts.

## Discussion

For a long time, pneumonia caused by *T. gondii* has been overlooked as a severe problem in immunocompetent hosts and thus few studies have been concerned with the disease. However, more and more reports have indicated that toxoplasmic pneumonia can be frequently found in individuals with normal immune function including both humans [12]–[17] and animals [18]–[21]. It was considered that rats, like humans, have a higher level of resistance to *T. gondii* infection in comparison to other mammals such as mice, guinea pigs and hamsters which all show a higher degree of susceptibility to this parasite [50]–[53]. Therefore, rats are recognized as a good animal model for the understanding of human toxoplasmosis [52], [54]–[59]. Previous studies from our laboratory demonstrated that the higher expression and activity of iNOS and lower expression and activity of arginase in RPMs are strongly linked with the resistance to the *T. gondii* RH strain infection in resistant (rats) and susceptible (mice) species [49]. This highlights the mechanism of species specificity to pathogen infection at least in *T. gondii*. Furthermore, these studies demonstrated that individual variation in susceptibility, based on differences in inbred lines of rats, could also be linked to the balance of expression of iNOS and arginase-1 [49]. Although RPMs were considered to be resistant to *T. gondii*, particularly the RH strain, interestingly, we have found that RAMs are much more susceptible to this parasite. This phenomenon was also reported by Chinchilla et al. [29] and Badger et al. [27] but the mechanism was not known. They did, however, report that interferon-γ was found to be involved in activating rat alveolar macrophages which in turn appeared to induce antimicrobial activity against *T. gondii in vitro* [60]. As far as we can ascertain, no other studies have investigated this phenomenon more recently. A key question that has not been addressed is the reason why RPMs show different susceptibility levels to RAMs during *T. gondii* RH strain infection. Previous studies [49] showed that iNOS and arginase-1 expression are strongly linked to resistance and susceptibility by peritoneal macrophages. This raises the question as to whether the expression and activity levels of iNOS and arginase-1 differ between RAMs and RPMs and could therefore account for differences in susceptibility. Our results clearly demonstrate that, in comparison to RPMs, there is a much lower level of expression of iNOS (and, consequently, a lower production of NO) but higher expression levels of arginase-1 (and, consequently, more recorded urea) in RAMs (Figure 3 and Figure 4). This could explain why the *T. gondii* RH strain grows well in RAMs but not in RPMs. In comparison with the peritoneal macrophages of mice, which are also highly sensitive to infection with *T. gondii*, RAMs also produce less NO. The presence of high levels of NO is considered to confer great benefit in preventing the growth of *T. gondii* and the consequent destruction of host cells [49]. This study shows a strong link between the balance of iNOS and arginase-1 activity in RAMs and their higher susceptibility to *T. gondii* than RPMs *in vitro* and *in vivo*. As far as we can ascertain, this has not been reported before and few studies have considered the interactions between *T. gondii* and RAMs. The presence of the phylogenetically unrelated parasite, *Trypanosoma lewisi*, has been shown to have an immunosuppressant effect on *T. gondii* infection in RAMs [61]. The mechanism for this is not known but may be triggering key generic pathways that suppress the proliferation of *T. gondii*. A recent study, using indoleamine 2,3-dioxygenase (IDO) gene knockout mice, has shown that increased IDO attenuates acute toxoplasmic infection in lung tissue [62]. This enzyme, which depletes local stocks of L-tryptophan is stimulated by effectors such as interferon-γ. Previous studies have reported associations between NO synthesis, interferon-γ and reduced microbial activity [63], suggesting that the roles of these molecules may also be linked to the regulation of *T. gondii* proliferation. More research is required to explore such associations.

An important question is why different expression levels of iNOS and arginase-1 exist in these cells from the same body?

It is well known that the lungs are more sensitive and prone to be injured by invading microorganisms, dust particles, harmful chemicals and autoimmune damage. Alveolar macrophages, therefore, are required to remain in a quiescent state to avoid damaging other pneumocytes by expressing minimal quantities of inflammatory cytokines and possessing lower levels of phagocytic activity [28], [64]. In fact, alveolar macrophages play a compensating role in the regulation of the immune defense in mammals. The adaptive immunity is suppressed by alveolar macrophages to prevent nonselective destruction of normal pneumocytes by secreting Th2 cytokines, such as transforming growth factor-β (TGF-*β*), interleukin-4 (IL-4), interleukin -10 (IL-10) and other small molecules [64]–[66]. Studies have shown that iNOS and arginase-1 are tightly regulated by Th1 cytokines (IFN-γ and TNF-α) and Th2 cytokines (IL-4 and IL-13) respectively [37], [67], [68]. In addition, the L-arginine metabolic pathway is very important for host defense and pathogen infection [69], [70]. Therefore, we presume that the lower expression and activity of iNOS and higher expression and activity of arginase-1 are highly correlated with Th1/Th2 cytokines regulated by alveolar macrophages in the quiescent state. However, other activators of arginase-1 are recognized such as STAT3 signaling cytokines IL-6, GCSF, and IL-10 [71]. Furthermore, it has been reported that an interaction between the *T. gondii* ROP16 protein kinase and the STAT3 and STAT6 pathways results in an increase in arginase-1 expression [72]. It is possible, therefore, that the *T. gondii* ROP protein kinase systems are also involved. This quiescent state and immunological down regulation in peritoneal macrophages were not found despite the fact that it is generally thought that both macrophage types originate from the blood mononuclear phagocyte system or bone marrow [73]. Recent studies have shown that some macrophages may develop from sources other than these traditional origins [74] – perhaps RPMs and RAMs are derived from a different source. Moreover, other differences have been reported in phenotype and function of these two macrophage types (peritoneal cavity and lung tissue) such as O_2_ metabolism, cell surface antigens and modulation of immune cell function [28], [64], [75]–[78]. Therefore, we suggest that the differences between phenotype and function of RAMs and RPMs may also be related to the differences in the L-arginine metabolic pathway.

In the L-arginine metabolic pathway, the competition for L-arginine by iNOS and arginase-1 results in an opposing status in relation to NO and urea (i.e. either higher NO and lower urea or lower NO and higher urea) in macrophages or possibly other types of cells [44], [45], [49]. There is a great deal of evidence to demonstrate that non-activated RPMs display a higher capacity to catalyze L-arginine to NO by means of higher expression of iNOS resulting in a lesser production of urea (or polyamines) [49], [79], [80]. It is well documented that the competition for arginine between iNOS and arginase affects the outcome of *T. gondii* infection [44], [45], [49] and our study clearly replicates this association when comparing RPMs and RAMs.

The marked differences in susceptibility to *T. gondii* infection between mice and rats [49] are clearly genetically determined by comparison of inbred mouse strains with inbred rat strains. Additionally, it was clearly demonstrated, by genetic crosses, that differences in infection susceptibility in RPMs from different rat inbred strains are genetically controlled [49]. The differences in susceptibility between RPMs and RAMs are an interesting genetic question. The fact that the RPMs and RAMs are derived from the same inbred rat strains, combined with the fact that different inbred rat strains also exhibit the same phenomenon, leads to the conclusion that differences in susceptibility (and arginine metabolism) are determined epigenetically. This raises interesting questions as to which host genes determine susceptibility to *T. gondii* infection and how host epigenetic mechanisms determine differences in susceptibility in different tissues. Our data suggest that the iNOS and arginase-1 genes are central to these questions.

In addition to host mechanisms which determine susceptibility or resistance to *T. gondii* infection, there are clearly parasite derived mechanisms [81]. In this study, strain RH was used as the model parasite due to the very clear differences in responses in rats and mice. RH is, however, a type I strain and is highly virulent, particularly to mice and this strain type is not the predominant type found in humans which tend to be Type II strains. Type II strains are much less virulent in mice and further work would be required to investigate the effect that these strains have on the arginase/iNOS balance in both mice and rats. Recent studies are beginning to shed light on the mechanisms of virulence derived from the parasite in mice and these are thought to involve the ROP2 family of protein kinases [82]. However, little is currently known about these processes in humans. An important future area of research should be aimed at linking the mechanisms determining virulence in the parasite with corresponding host mechanisms to resistance and susceptibility.

In summary, the characteristics of RAMs infected with *T. gondii* are clearly defined and these cells are susceptible to *T. gondii* RH strain infection both *in vitro* and *in* *vivo*. It is clear that the native biological characteristics in macrophages from the lungs and peritoneal cavity of rats are strongly linked with their resistance or susceptibility to *T. gondii* infection. Our results have demonstrated that the lower expression and activity of iNOS but higher activity of arginase (and higher polyamine levels) in RAMs are linked to the susceptibility to *T. gondii* infection. The question of how these results from rats relate to humans requires further research. The mechanisms of action of human macrophages to microbial challenge differs from that in rats and mice. It is widely reported that iNOS and arginase are not expressed under similar stimulation conditions in human macrophages *in vitro* [83], however, *in vivo*, they have been shown to express iNOS and arginase-1 and produce NO. Future studies need to address these differences. Nevertheless, an understanding of the interactions between *T. gondii* and host macrophages promises to provide great benefit to our understanding of the pathogenesis and mechanisms of pulmonary toxoplasmosis in humans and domestic animals.

## Materials and Methods

### Ethics Statement

All animal experiments were conducted using the guidelines provided by the Laboratory Animal Use and Care from the Chinese CDC and the Rules for Medical Laboratory Animals (1998) from the Ministry of Health, China. All protocols for animal use in this work were approved by the Laboratory Animal Use and Care Committee of Sun Yat-Sen University under the license number 2012CB53000.

### Animals and Parasites

Sprague Dawley (SD), Wistar rats and BALB/c mice were purchased from the Laboratory Animal Center of Sun Yat-Sen University; Brown Norway (BN), Fischer 344 (F344) and Lewis rats were purchased from Vital River Laboratories (Beijing, China). All rats (6 to 8 weeks old, weight 150∼200 g) and mice (6 to 8 weeks old, weight 18∼25 g) were certified to be free of specific pathogens when entering the laboratory and maintained with sterile distilled water and commercial food.

The *T. gondii* strain RH-GFP, kindly provided by Dr. Xue-Nan Xuan of the Obihiro University of Agriculture and Veterinary Medicine, Japan, was generated as previously described [84]. For the purification of tachyzoites, host cell debris and *T. gondii* were harvested from the peritoneal cavities of infected BALB/c mice by injection of cold D-Hanks solution three days after infection. The solution containing *T. gondii* was centrifuged at 50×g for 5 min at 4°C to discard host cells and fragments. The resulting supernatant was then centrifuged at 1350×g for 10 min at 4°C, and then was suspended in RPMI-1640 medium (GIBCO, USA) with 10% FBS and the parasites counted.

### Isolation and Cultivation of Peritoneal and Alveolar Macrophages

Rats or mice, anaesthetised by carbon dioxide (CO_2_), were injected intraperitoneally with 15 mL (rat) or 5 mL (mouse) of cold calcium and magnesium-free Dulbecco’s phosphate buffered saline (D-Hanks) containing 100 U of penicillin and 100 µg of streptomycin per ml. To obtain the peritoneal macrophages, 11–14 ml (rat) and 3–4 ml (mouse) of peritoneal cavity fluid was collected from each animal. For each experiment, the cells from 1–2 rats, or 3–4 mice, were pooled and then cultivated as described below.

Resident alveolar macrophages were obtained from the lungs *in situ* by the methods described by Myrvik et al. [85] and Catterall et al. [5]. Briefly, rats were injected peritoneally with 2.5% hydral at 50 mg/kg and a sterile #26 pipe conveying fluid was used. The lungs were lavaged with cold (4°C) D-Hanks containing 100 U of penicillin and 100 µg of streptomycin per 1 ml with a 10 ml plastic syringe. This process was repeated until a total of 50 mL lavage fluid was collected. For each experiment, cells from 5–6 rats were pooled and then cultivated as described below.

The lavage fluid or harvested peritoneal macrophages were centrifuged at 250×g, for 10 min at 4°C. The pellet was resuspended in 5 mL of RPMI-1640 medium (GIBCO Laboratories, USA) with 10% fetal bovine serum (FBS; GIBCO Laboratories, USA) and penicillin (100 U/mL) and streptomycin (100 µg/mL). In all experiments, the viability of cells was higher than 95 percent as measured by the trypan blue exclusion test. More than 92% of rat or mouse peritoneal lavage cells and >95% of rat alveolar lavage cells were assessed to be phagocytes as determined by the uptake of neutral red. Cells were counted and seeded in a 96 well plate (2×10^5^ cells/well) or 6 well plate (2×10^6^ cells/well) for 2 h at 37°C, 90% humidity and 5% CO_2_. After 2 h, wells were washed with sterile DPBS (pH 7.2) to remove non-adherent cells and fresh culture medium was added. Macrophages were incubated with or without lipopolysaccharide (LPS; 10 µg/ml, Sigma, St. Louis, USA) plus IFN-γ (100 U/ml; Sigma, USA) or with the NOS specific inhibitor Nω –nitro-L -arginine methyl ester (L-NAME; 10 mM; Sigma, St. Louis, USA). Cells were then cultured overnight and then used for further experiments.

### Parasite Infection and Proliferation

Rat alveolar and peritoneal macrophages isolated and cultivated, as described above, were challenged with *T. gondii* tachyzoites at the ratio of 1∶1 (*T. gondii*/macrophage) only once. Extracellular *T. gondii* were then washed out 1 hr after incubation with the cells. This time point was defined as 1 hr for the start of the experiment. Thereafter, the results were observed under an inverted fluorescence microscope or stained with DAPI at each desired time point. When time point analysis was carried out (e.g. 1 hr and 24 hrs), the same population of cells was sampled. Infection results from 96 well plates were observed and analyzed by merged photographs of normal and fluorescence pictures. Six well plates and cell slides were used for the DAPI experiments. All the results were observed by fluorescence microscopy using a 350 nm exciting wavelength and 495 nm emitting wavelength. The number of *T. gondii* within the cells and the infected cells were counted in 100 macrophages which were randomly selected from different fields of view at least 3 times and an average was determined. Samples from 1 hr to 24 hrs in infection experiments were observed in independent 96 or 6 well plates respectively. These experiments were performed no less than twice.

### Measurement of Production of Nitric Oxide and Arginase Activity

Nitrite content as a reflection of NO production was determined by the Griess reaction as previously described [86]. Briefly, 100 µl supernatant or standard solution (NaNO_2_) were incubated with 100 µl of Griess reagent (0.5% sulfanilamide, 0.05% naphthyldiamine dihydrochloride in 5% H_3_PO_4_) for 10 min. The plates were read at 550 nm in an ELISA reader (Multiskan MK3, Thermo Labsystems, Finland). All experiments were carried out in triplicate.

Arginase activity in purified macrophages was measured by a colorimetric method as described [87]. Briefly, 10 mM MnCl_2_ and 0.5 M L-arginine were successively added to macrophage lysates for 1 hr at 37°C. The reaction was stopped by the addition of an acid solution (H_2_SO_4_:H_3_PO_4_:H_2_O = 1∶3∶7) and the urea generated by arginase was analyzed by addition of α-isonitrosopropiophenone at 100°C for 45 min. The colored product was quantified by absorption at 550 nm in an ELISA reader. Arginase activity was determined as the amount of urea produced from total protein in the peritoneal and alveolar macrophages.

### RT-PCR Analysis

Total RNA from treated and nontreated macrophages was extracted using Trizol Reagent (Invitrogen, Carlsbad, USA) according to the manufacturer’s instructions. Total RNA was reverse transcribed to cDNA using a set of oligo (dT) primer and SuperScript™ III First-Strand Synthesis System according to the instructions by the manufacturer (Invitrogen, Carlsbad, USA). cDNA (1 µg) was used as a template for amplifying the iNOS, arginase-1 and GAPDH (used as internal standard) genes by PCR using the following primers: rat-iNOS, 5′-CTA CCT ACC TGG GGA ACA CCT GGG-3′ and 5′-GGA GGA GCT GAT GGA GTA GTA GCG G-3′ 442 bp; mouse-iNOS, 5′-GCC TCG CTC TGG AAA GA-3′ and 5′-TCC ATG CAG ACA ACC TT-3′, 499 bp; arginase-1, 5′-AAG AAA AGG CCG ATT CAC CT-3′ and 5′-CAC CTC CTC TGC TGT CTT CC-3′, 201 bp; GAPDH, 5′-AAT GCK TCC TGY ACC ACC AAC TGC-3′ and 5′-TTA GCC AWA TTC RTT GTC RTA CCA GG-3′, 513 bp. For semi-quantitative PCR, the cycling conditions were: 94°C for 1 min, 60°C for 1.5 min and 72°C for 1.8 min. For rat-iNOS, mouse-iNOS and arginase-1, 27 cycles were used, but for GAPDH, only 20 cycles were used. Amplified DNA products were separated on 1% agarose gel and were photographed using an electronic documentation system (Biostep, Germany) after staining with ethidium bromide.

### Western Blotting

Cells were lysed in SDS loading buffer, fractionated in SDS-PAGE and transferred onto immunoblot polyvinylidene difluoride membrane (Pall, USA). The membrane was probed using the rabbit polyclonal iNOS antibody (Thermo, USA ) and rabbit polyclonal arginase-1 antibody (Santa Cruz, USA). *β*-actin was stained with antibody (NOVUS, USA) as a sampling control. Horseradish peroxidase-labeled secondary antibodies (Cell Signaling, USA) and BeyoECL Plus Detection Kit (Beyotime, China) were used for antibody detection.

### Infection of *Toxoplasma* in Rat Alveolar Macrophages *in vivo*

SD or Wistar rats were randomly divided into 3 groups (6 rats in each group) and were infected by syringe into the lung tissue and peritoneal cavity with 2×10^7^ tachyzoites of RH strain per rat. The rats in the control group were injected with PBS only. Rats from each group were anaesthetised and dissected 24 hrs after infection. Brains, hearts, lungs, livers and spleens were removed from the rats. A part of each organ was smeared with a tip and then stained with Diff after being dried and fixed in methyl alcohol. The rest of the tissues of the rats were fixed in 10% polyphosphate formalin for not more than 48 hrs and were then prepared for examination by immunohistochemistry with anti-*T. gondii* antibody (Abcam, USA) to analyze infection in rat alveolar macrophages.

### Statistical Analysis

Results are expressed as a mean ± SEM. In the case of the *in vitro* macrophage studies, experiments were conducted in triplicate using the same sample at different time points. *In vivo* studies were conducted on 6 rats per study group. Statistical differences were designated by * (*p*<0.05), ** (*p*<0.01) and *** (*p*<0.001) or in some cases where multiple comparisons are used # (*p*<0.05), ## (*p*<0.01) and ### (*p*<0.001). Multiple data comparisons were derived by one-way ANOVA by SPSS 17.0 software (SPSS Inc., Chicago, USA).
